# Blocking of PI3-kinase beta protects against cerebral ischemia/reperfusion injury by reducing platelet activation and downstream microvascular thrombosis in rats

**DOI:** 10.1038/s41598-023-29235-2

**Published:** 2023-02-04

**Authors:** Qiong Cheng, Min Wang, Rong Jin, Guohong Li

**Affiliations:** 1 Department of Neurosurgery, Penn State Hershey Medical Center, Hershey, PA 17033 USA; 2 Key Laboratory of Neuroregeneration of Jiangsu and Ministry of Education, Nantong University, Nantong, 226000 China

**Keywords:** Medical research, Neurology

## Abstract

Phosphoinositide 3-kinase beta (PI3Kβ) plays an important role in platelet activation and thrombosis, but its role in stroke pathology remains unknown. In this study, we investigated whether inhibition of PI3Kβ protects against cerebral ischemia/reperfusion (I/R) injury by preventing circulating platelet activation and downstream microvascular thrombosis. We used a rat intraluminal filament model of transient middle cerebral artery occlusion (tMCAO) because the rapid restoration of cerebral blood flow to the ischemic area in both tMCAO and endovascular thrombectomy provides clinical relevance for this model. The results showed that TGX221, a selective PI3Kβ inhibitor, treatment immediately before the onset of reperfusion dose-dependently reduced infarct volume and improved neurological function. The protective effects were associated with blocking platelet activation and thrombotic response, thereby reducing downstream microvascular thrombosis, and maintaining reperfusion efficiency. These results suggest that PI3Kβ might be a promising target for treating downstream microvascular thrombosis induced by cerebral I/R injury and offer a novel adjunctive treatment to improve reperfusion therapy for acute ischemic stroke.

## Introduction

Ischemic stroke is a leading cause of death and permanent disability worldwide^1^. Reperfusion therapy is the most effective treatment for patients with acute ischemic stroke (AIS). While thrombolysis with intravenous tissue-type plasminogen activator (IV tPA) is the only the FDA approved treatment for patients with AIS, mechanical thrombectomy (MT) developed over the past decade has revolutionized reperfusion therapy for AIS patients with large vessel occlusion^2^. However, about one-third of AIS patients do not show clinical improvement possibly because of incomplete microvascular reperfusion despite successful recanalization of the occluded large artery^2,3^. Downstream microvascular thrombosis is proposed to be a key contributing factor to incomplete microvascular reperfusion after reperfusion therapy for AIS^4,5^, and no effective adjunctive treatments are available for preventing downstream microvascular thrombosis in AIS patients after reperfusion therapy. Although current antiplatelet drugs (e.g. aspirin, clopidogrel, and glycoprotein IIb/IIIa receptor antagonists) have been tested as an adjunct to reperfusion therapy for stroke victims, however, no clinical trials have yet demonstrated reliable and safe antiplatelet treatments, mainly due to increased risk of intracranial hemorrhage^6,7^. Thus, there is an urgent need for developing effective adjunctive approaches to current reperfusion therapy for AIS patients without unwanted extension of bleeding time.

Phosphoinositide 3-kinases (PI3Ks) are a family of lipid kinases divided into three classes (I, II, and III) with distinct structures and functions^8^. Class I PI3Ks, the most studied class, contain four isoforms, the three class IA (PI3Kα, PI3Kβ, and PI3Kδ) and the sole class IB (PI3Kγ), named by their catalytic subunit (p110α, p110β, p110δ, p110γ)^8^. PI3Kα and PI3Kβ are ubiquitously expressed and knocking out either gene in mice results in an embryonic lethal phenotype, whereas PI3Kγ and PI3Kδ expression is low in most cells but found at high levels in hematopoietic cells^9^. Using genetic manipulation as well as pharmacological approaches, an increasing number of studies have been conducted to define overlapping and non-redundant functions of different PI3K isoforms. Previous studies have revealed important but differential roles for specific class I PI3K isoforms in platelet activation and thrombosis^10^. Among various PI3K isoforms, PI3Kβ has emerged as a crucial mediator of platelet aggregation and thrombosis^11–14^, while PI3Kγ acts as a crucial mediator of immune and inflammatory responses^15,16^. Thus, PI3Kβ and PI3Kγ have emerged as anti-thrombotic and anti-inflammatory targets, respectively, for drug discovery^17^. Targeting thromboinflammation (thrombotic and inflammatory processes) is emerging as a novel strategy for the treatment of AIS^18^. We have previously shown that genetic deletion of PI3Kγ reduces brain inflammation and oxidative stress in acute experimental stroke in mice^19^. However, the role of PI3Kβ in stroke pathology has not yet been investigated. In this study, we investigated whether PI3Kβ contributes to downstream microvascular thrombosis and ischemic brain damage in a rat stroke model of focal cerebral ischemia followed by reperfusion.

## Methods

Animal experiments were performed following the National Institutes of Health Guide for the Care and Use of Laboratory Animals in compliance with ARRIVE guidelines^20^. Use of Laboratory Animals and the animal protocols were approved by the Institutional Animal Care and Use Committee (IACUC) at Penn State College of Medicine. Data supporting the findings of this study are available from the corresponding author upon reasonable request. All data analyses were performed in a blinded manner.

### Transient intraluminal middle cerebral artery occlusion (tMCAO) model

Adult male Sprague–Dawley rats (weighing 280–320 g, Charles River Laboratories, Wilmington, Massachusetts, USA) were subjected to 2 h of tMCAO followed by reperfusion, as we described previously^21^. Briefly, animals were anesthetized with isoflurane (5% for induction and 2% for maintenance). A silicon-coated nylon monofilament (#503934PK5Re, Doccol Corp.) was advanced through the external carotid artery into the internal carotid artery until lodging proximal to the origin of the middle cerebral artery (MCA). Regional cerebral blood flow (rCBF) in the MCA territory (2 mm posterior and 5 mm lateral to the bregma) was monitored with a Laser doppler flowmetry (MSP300XP; ADInstruments Inc). Only animals that showed sustained ischemia to less than 15% rCBF of preischemic baselines were included in this study. During the procedure, body temperature was maintained at 37.0 ± 0.5 °C using a feedback-regulated heating pad system. PE-50 catheters were placed into the vein for drug administration.

### Experimental protocols

To test the dose–response of TGX-221, rats were subjected to tMCAO and randomly assigned into the following 4 groups: vehicle [ethanol/cremaphor/N,N-dimethylacetamine/water (10/10/10/70%)] and varied doses of TGX221 including 0.3, 1, and 3 mg/kg. The range of doses was based on previous publications for animal experiments^22,23^. TGX221 or an equal volume of vehicle was administrated at 2 h and repeated at 24 and 48 h after stroke onset using a syringe infusion pump for 2 min. The modified Bederson score, used to determine global neurological function, was performed by a blinded investigator before and 72 h after stroke, as we described previously^21^. The grip strength test used to determine the motor function and deficit after stroke was also performed with an Animal Grip Strength System (Bioseb, Pinellas Park, FL USA). Animals were euthanized with CO2 at 72 h after stroke. For randomization, the web tool www.randomizer.org was used. Animals were randomly assigned to each group via random numbers generated on an Excel spreadsheet. Based on the dose–response assessment, the optimal dose of TGX221 (3 mg/kg) was chosen for further studies.

### Infarct volume and hemorrhage

The infarct volume was measured in TTC-stained coronal sections at 72 h after stroke. Then, the sections were homogenized and the hemoglobin levels were assessed by a spectrophotometric assay using Drabkin reagent (SigmaAldrich) as described previously^21,24^.

### Immunohistochemistry

At 24 h after stroke, animals were euthanized with CO2. Brains were removed and fixed in 4% paraformaldehyde. A standard paraffin block was obtained from the center of the ischemic lesion (bregma − 0.4 to − 1.4 mm). 5 μm thick coronal sections were cut. Every tenth coronal section for a total of 5 sections was used for immunohistochemistry. Double immunostaining was used to detect fibrin and platelets deposited within brain microvessels (marked by endothelial barrier antigen [EBA] staining) as described previously^24,25^. The section was incubated with primary antibodies overnight at 4 °C and then incubated with fluorescent secondary antibodies for 1 h at RT. Isotype controls were used as negative controls. To maintain consistency within and between animal groups, two predefined fields within the cortical ischemic boundary zone (IBZ) were selected as regions of interest (ROI), as shown in Fig. 2E.

### Cerebral microvascular patency

Cerebral microvascular patency was assessed by the FITC-dextran labeled vessels, as we described previously^24^. Briefly, 1 ml of FITC-dextran (50 mg/ml, 2 × 10^6^ MW, Sigma-Aldrich) was injected intravenously and allowed to circulate for 5 min. Then, the animals were euthanized by cervical dislocation. The brain samples were collected and 100 µm coronal sections were cut using a vibratome. Five (5) coronal sections (at the bregma level: + 0.2, − 0.4, − 1.0, − 1.6, − 2.0 mm) of each brain were selected for the measurement of cerebral perfusion. The ROI was defined as described above. To quantify the microvascular patency, the intensity of FITC-dextran was quantified using NIH Image Software. Data are presented as a percentage of the fluorescence intensity in the ipsilateral hemisphere compared to sham controls.

### Platelet activation and platelet-leukocyte aggregates

At 24 h after stroke, the blood sample was drawn from the abdominal aorta in 1:7 (v/v) of acid–citrate–dextrose buffer under deep anesthesia. For the evaluation of platelet activation, the surface expression of P-selectin and the ability to bind FITC-fibrinogen were measured. Samples were co-stained with the APC mouse anti-rat CD42d (1:200, RPM.4) and PerCP/Cy5.5 mouse anti-rat CD62P (1:100, RMP-1) for 20 min at RT, or incubated with FITC-conjugated rat fibrinogen (innovative-research,100 μg/ml) for 30 min at 37 °C. After that, the reaction was stopped by adding 1 ml PBS, and the samples were analyzed on the BD Accuri™ C6 flow cytometer. For detection of platelet–leukocyte aggregates (PLA) formation, samples were co-stained with anti-rat CD42d (1:200, RPM.4), anti-rat granulocytes (1:100, RP-1), anti-rat CD11b (1:100, ED8) and anti-rat CD45 (1:100, OX-1) at RT for 20 min. Isotype-matched control antibodies were used as negative controls. Then, the red blood cell was lysed with red blood cell lysis buffer. Before staining, all samples (10ul whole blood) were diluted 1:10 with Flow Cytometry Staining Buffer (eBioscience) containing rat Fc block anti-rat CD32 and incubated for 10 min at RT.

### Platelet protein extraction and western blot

The anticoagulated blood sample was centrifuged at 220×*g* for 20 min, and the platelet-rich plasma (PRP) was collected and transferred to a new tube for centrifugation at 480×*g* for 20 min at RT. The supernatant (plasma) was collected and stored at − 80 °C for further ELISA assay to detect the concentration of TXB2 (ADI-900-002, Enzo Life Sciences). Then, suspend the pellet (pure platelet) with 10 volumes of Thermo Scientific M-PER Mammalian Protein Extraction Reagent containing protease/phosphatase inhibitor Cocktail (Cat:5872, Cell Signal). The suspension was subjected to three cycles of sonication of 5 s each and centrifuged at 13,000×*g* at 4 °C for 10 min. 50 µg total protein per lane was separated by SDS-PAGE and transferred onto polyvinylidene difluoride (PVDF) membranes. The membranes were incubated in blocking buffer (5% w/v nonfat dry milk in Tris-buffered saline, 0.05% Tween-20 [TBS-T]) for 1 h at RT, and then incubated with primary antibodies overnight at 4 °C. Next, the membranes were incubated with horseradish peroxidase-conjugated secondary antibody at RT for 1 h.

### Platelet aggregation assay

PRP was collected as above described and platelet-poor plasma (PPP) was obtained from the remaining sample by centrifugation at 1500×*g* for 30 min. The platelet concentration was adjusted to 5 × 10^8^/ml by mixing PRP and PPP. Platelet aggregation was measured using an optical model 700 aggregometer (Chrono-Log) at 37 °C under constant stirring (1200 rpm). PRP (250 ul per tube) was incubated without or with different doses of TGX221 at RT for 30 min and then aggregation was induced with 10 µmol/l ADP (Cat: 20398-34-9, Sigma). Aggregation was measured as changes in light transmission for 6 min at 37 °C.

### Tail bleeding time assay

Tail bleeding time was measured in rats using a modified tail-cutting method^24^. At 24 h after stroke, animals were anesthetized with isoflurane and placed on a feedback-regulated heating pad system. Bleeding times were measured by the transaction of the tail, 2 mm from the tip using a disposable surgical blade. The cut was dabbed with filter paper every 15 s until the paper was no longer stained red with blood. Bleeding time was then taken as the time when the blood stopped flowing from the cut tail.

### Statistical analysis

All results were expressed as mean ± standard deviation (SD). GraphPad Prism 8.0 software package was used for statistical analysis. Unless otherwise indicated, multiple comparisons were made using a 1-way analysis of variance (ANOVA) followed by the Bonferroni post hoc test. If only 2 groups were compared, an unpaired, 2-tailed Student t-test was applied. P < 0.05 was considered statistically significant.

### Ethical approval and consent to participate

All animal experiments in this study have been approved by the Institutional Animal Care and Use Committee of Penn State University College of Medicine in accordance with the National Institutes of Health (NIH) Guide for the Care and Use of Laboratory Animals.

## Results

### Inhibition of PI3Kβ ameliorates acute ischemic stroke injury

**Figure 1:**
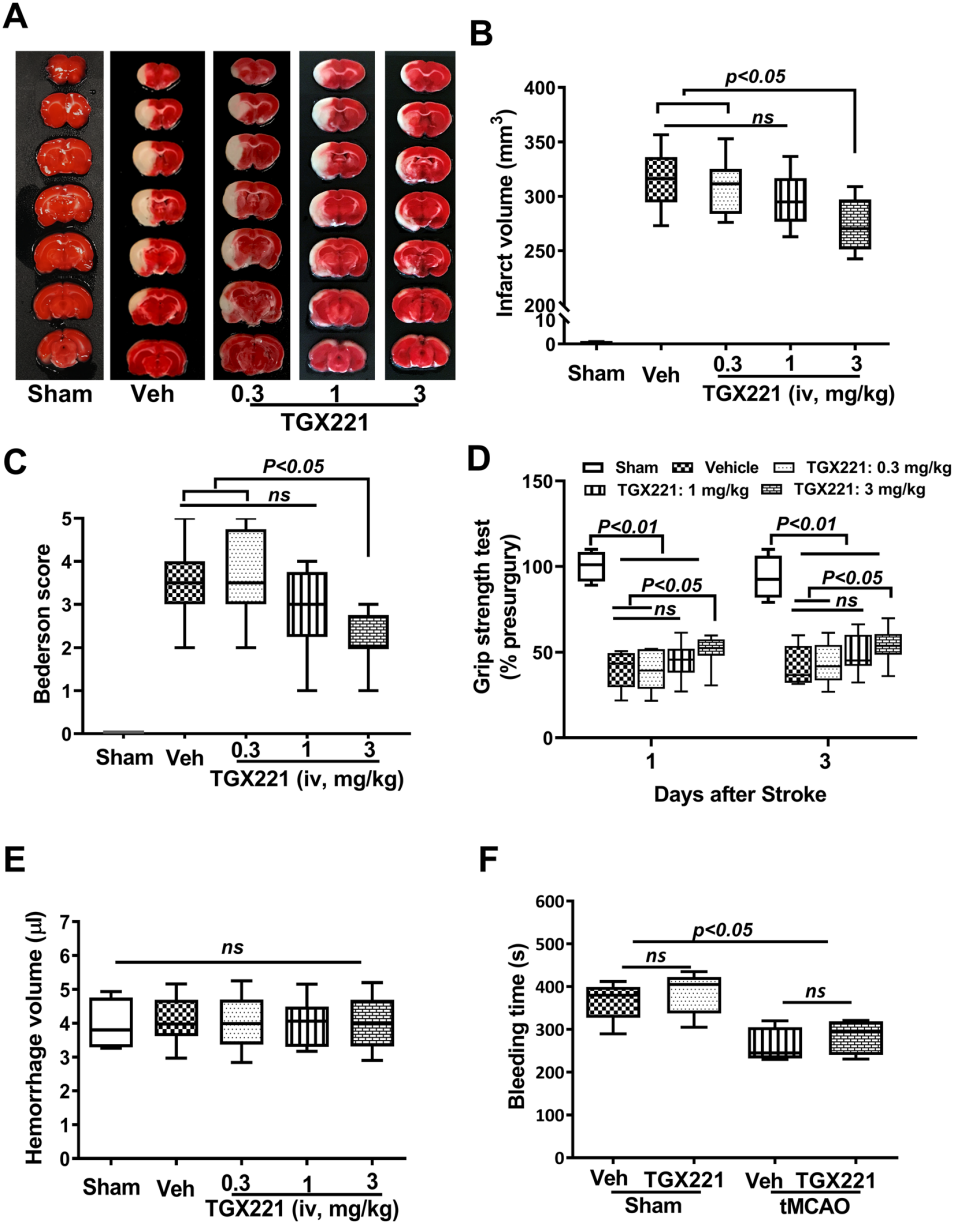
Inhibition of Pi3K-β improves acute stroke outcomes. (**A**) Representative TTC staining; (**B**) infarct volume; (**C**) Bederson score; (**D**) Grip strength test, and (**E**) Hemorrhage volume. n = 8 per group; All animals were subjected to 2 h tMCAO followed by reperfusion for 72 h. Different doses of TGX221 were given at 2 h after stroke and repeated at 24 and 48 h. (**F**) Tail bleeding time, n = 5/group, animals were subjected to 2 h tMCAO followed by reperfusion for 24 h. *ns* not significant. Non-parametric Bederson scores were compared by Kruskal–Wallis test with post-hoc Dunn corrections.

Acute stroke injury was assessed on day 3 after tMCAO. Our results showed that treatment with PI3Kβ inhibitor, TGX-221, decreased brain infarction and neurological deficit in a dose-dependent manner. Infarct volume was significantly reduced in stroked rats treated with a high dose of TGX221 (3.0 mg/kg, p < 0.05), whereas lower doses of TGX-221 (0.3 mg/kg and 1.0 mg/kg) had no significant effect (p > 0.05) when compared to the vehicle-treated group (Fig. 1A,B). Importantly, the smaller infarcts translated into better neurological outcome. The stroked rats treated with 3.0 mg/kg TGX-221 showed significant improvement in the Bederson score and grip strength test when compared to either vehicle- or 0.3 mg/kg TGX221-treated groups (Fig. 1C,D). No significant differences in brain damage and neurological deficits were observed between 0.3 and 1.0 mg/kg TGX221-treated groups. Moreover, no significant differences in the brain hemorrhage volume (Fig. 1E) and the tail bleeding time (Fig. 1F) were observed between vehicle- and all TGX221-treated groups, indicating that therapeutic inhibition of PI3Kβ with TGX-221 did not increase the risk of bleeding after tMCAO in rats. Based on these data, TGX-221 at the dose of 3 mg/kg was used for further experiments.

### Inhibition of PI3Kβ reduces downstream microvascular thrombosis and improves microvascular reperfusion after tMCAO

**Figure 2:**
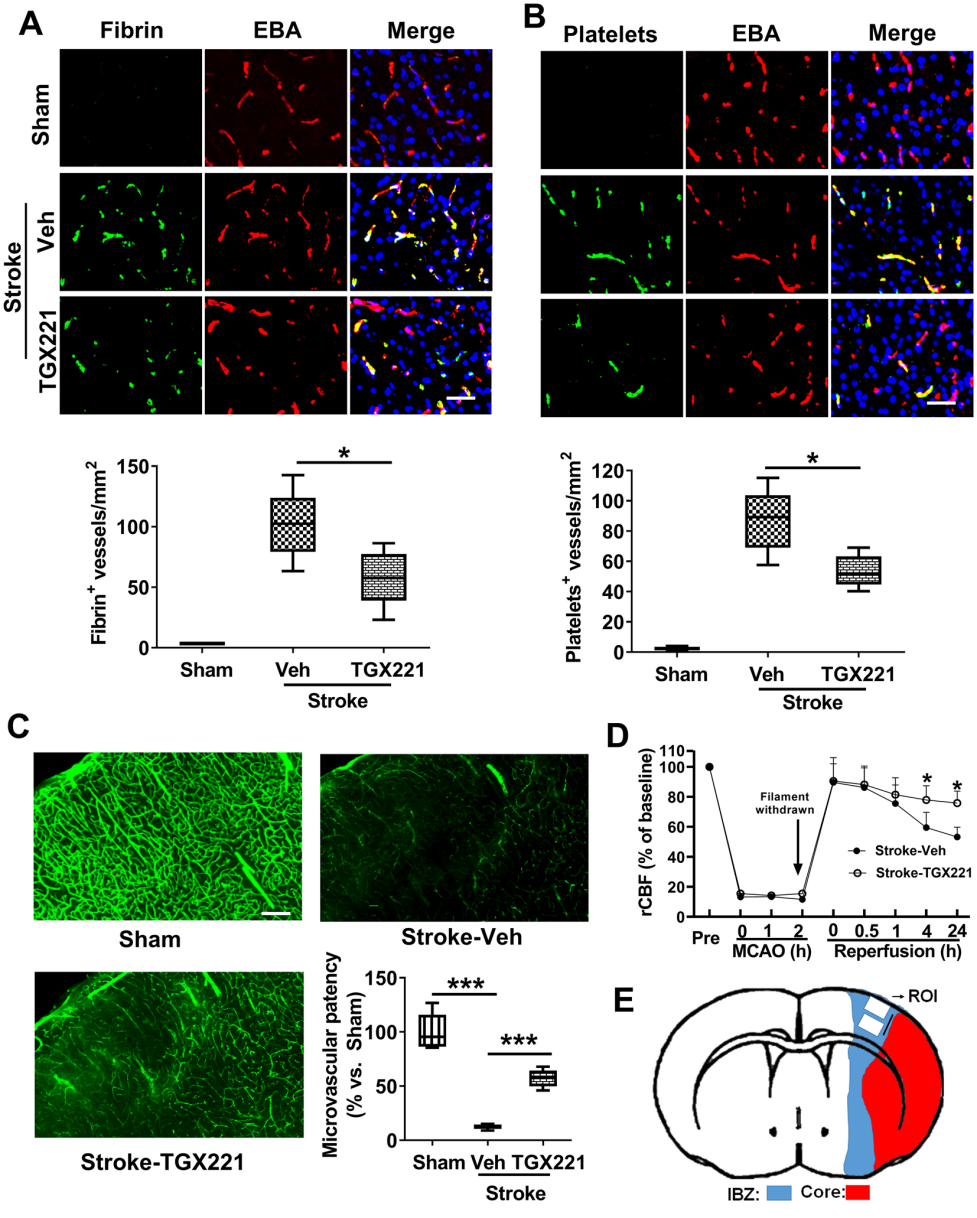
TGX221 treatment reduces microvascular thrombosis and improves patency. (**A,B**) Representative images of double immunofluorescence staining showing fibrin (**A**) or platelet (**B**) deposited within brain microvessels (marked by endothelial barrier antigen [EBA] staining). The number of double fibrin-positive (**A**) or thrombocyte-positive (**B**) vessels within microvessels in each ROI was counted, and the data were expressed as mean ± SD of double positive signals microvessels/mm^2^; *P < 0.05. N = 5/group. Scale bars = 50 μm. All animals were subjected 2 h ischemia followed by reperfusion for 24 h. (**C**) Representative images showing microvascular FITC-dextran perfusion in the indicated groups at 24 h after stroke. Scale bars = 100 μm. Quantitative data are expressed as the percentage of the microvascular area perfused with FITC–dextran in the ipsilateral hemisphere relative to sham control. n = 5/group. ***P < 0.001. (**D**) Changes of regional cerebral blood flow (rCBF). n = 5/group, *P < 0.05. (**E**) Schematic diagram showing the regions of interest (ROI) within the cortical ischemic boundary zone (IBZ).

To assess microvascular thrombosis, fibrin and platelet deposition in the brain microvessels were examined by double immunofluorescence staining. Our data showed that tMCAO caused extensive intravascular deposition of both fibrin (Fig. 2A) and platelets (Fig. 2B) in the brain microvessels, accompanied by a marked reduction in microvascular patency (Fig. 2C) at 24 h after tMCAO. The success of the induction of focal cerebral ischemia followed by reperfusion was verified by rCBF monitoring (Fig. 2D). Further, we found that the treatment with TGX-221 (3.0 mg/kg) profoundly reduced microvascular thrombosis and improved microvascular patency, thereby improving microvascular reperfusion after tMCAO (Fig. 2A–D).

### Inhibition of PI3Kβ attenuates platelet activation and prothrombotic response after tMCAO

No study to date has reported whether cerebral I/R alters PI3Kβ expression in the brain and blood cells. We initially examined PI3Kβ expression in the normal and ischemic brain (Suppl Fig. S1). Immunohistochemistry showed that constitutive expression of PI3Kβ was detected readily in neuron-like cells in the normal rat cortex and its level was not significantly altered at 24 h after cerebral I/R (Fig S1a). This result was consistent with Western blotting (Fig S1b). Of note, we did not observe immunopositive signals in other cell types both in the normal and ischemic brain.

**Figure 3:**
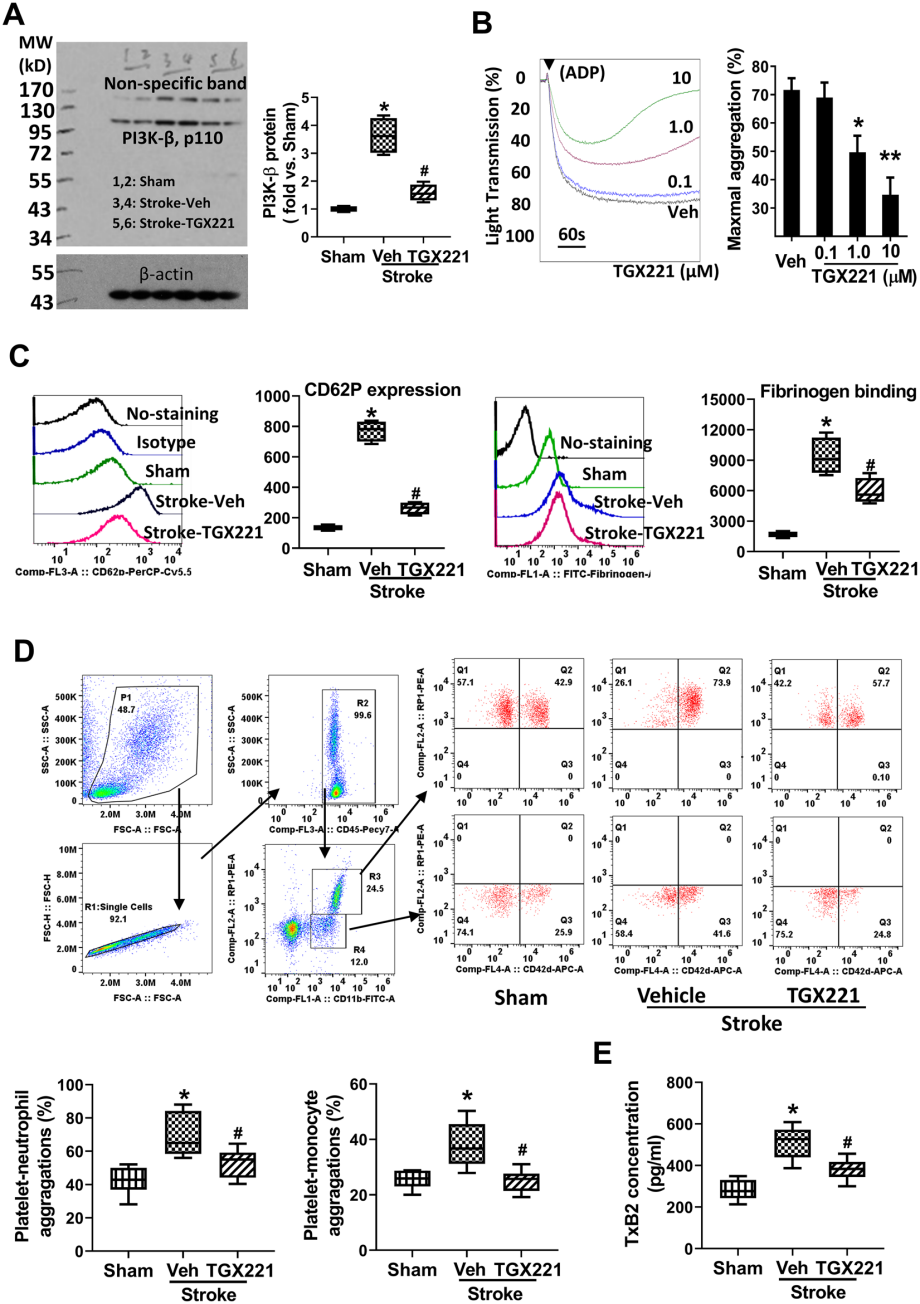
TGX221 treatment attenuates stroke-enhanced thrombotic response. (**A**) Representative images of western blots showing PI3Kβ protein levels in the platelets and semi-quantitative analysis of immunoblots. n = 4/group. *p < 0.05 vs. sham; #p < 0.05 vs. vehicle. Platelets were collected at 24 h after stroke. (**B**) Representative aggregation traces. Citrated PRP from normal rats were preincubated with different doses of TGX221 or vehicle for 30 min and then stimulated with 10 µmol/L ADP. Quantitative results are expressed as mean ± SD of three independent experiments. *p < 0.05 vs. Veh or 0.1 µm TGX221; **p < 0.01 vs. Veh or 0.1 µm TGX221. (**C**) Flow cytometry analysis of PerCP-Cy5.5-CD62p expression on platelets or FITC-Fibrinogen binding to platelets. Data are expressed as mean fluorescence intensity. n = 5/group. *p < 0.05 vs. sham; #p < 0.05 vs. vehicle. (**D**) Gating strategy for flow cytometric analysis of total leukocytes (R2, CD45 positive cells included in R1 single cells) in whole blood: neutrophils (R3) and monocytes (R4) were defined by their differential expression of CD11b and RP-1 (specific for rat granulocytes); and representative flow cytometric dot plots of platelet-neutrophil (CD42d + CD45 + CD11b + PR1 +) and –monocyte (CD42d + CD45 + CD11b + PR1−) aggregates in the indicated groups. APC-labeled CD42d (glycoprotein V) mAb was used as a specific platelet marker to identify platelet neutrophil/monocyte complexes. n = 5/group. *p < 0.05 vs. sham; ^#^p < 0.05 vs. vehicle. (**E**) ELISA assay of TXB2 concentration in plasma 24 h after stroke. N = 5/group. *p < 0.05 vs. sham; ^#^p < 0.05 vs. vehicle.

Next, we examined PI3Kβ expression in the platelets. Western blot analysis showed that the protein level of PI3Kβ expression in platelets was elevated significantly at 24 h after tMCAO, and this elevation was markedly blocked in the TGX221-treated group as compared to vehicle-treated group (The full images of western blot were available in Supple. Fig. S2) (Fig. 3A). To determine the role of PI3Kβ in platelet activation and aggregation, we first assessed the effects of TGX-221 on ADP-induced platelet aggregation in platelet-rich plasma (PRP) from normal rats. The results showed that the TGX-221 blocked ADP-induced platelet aggregation in a dose-dependent manner (Fig. 3B). Next, we assessed circulating platelet activation and platelet-leukocyte interaction, two known prothrombotic events during ischemic stroke. As expected, tMCAO induced platelet activation (determined by P-selectin expression and its ability to bind with fibrinogen) (Fig. 3C) and platelet-leukocyte aggregates (Fig. 3D) compared with the sham control, and the tMCAO-induced effects were profoundly reduced in the TGX221-treated group compared with the vehicle-treated group. In addition, we found that the TGX-221 treatment significantly reduced plasma levels of TXB2, a stable and inactive metabolite of Thromboxane A2 (TXA2) at 24 h after tMCAO (Fig. 3E).

## Discussion

The primary therapeutic goal for patients with AIS is to timely restore blood flow to the regions of brain that are ischemic but not yet infarcted. However, not all AIS patients benefit from reperfusion therapy. Despite successful recanalization of occluded large cerebral vessels, further infarct growth and neurological worsening, called ischemia/reperfusion (I/R) injury, can be observed^26,27^. The occlusion of the middle cerebral artery (MCA) and its branches account for ~ 70% of human strokes. Among several animal models of ischemic stroke, the transient MCAO model that affects the MCA territory is very close to the human situation and therefore widely used in experimental stroke research^28^. In this model, complete recanalization is achieved by the removal of the suture filament, while cerebral microvascular perfusion is incomplete, only about 50 ~ 70% restored to the baseline^28^. The rapid recanalization of the occluded MCA with incomplete reperfusion to downstream microvessels provides clinical relevance for this model to endovascular reperfusion therapy (mechanical removal of thrombi) for AIS patients^29^. In this study, the suture MCAO model was used to investigate the role of PI3Kβ in cerebral I/R injury.

It is widely accepted that activated platelets serve as critical modulators of I/R injury in various organs including brain. Experimental data (mostly using the suture MCAO model) show that circulating platelet activation and interactions with each other (homotypic aggregation) or with circulating leukocytes (heterotypic aggregation) contribute substantially to the pathophysiology of downstream microvascular thrombosis after cerebral I/R injury^30–32^. However, no clinical trials have yet demonstrated reliable and safe treatments with currently available antithrombotic drugs for AIS patients, mainly due to increased risk of brain hemorrhage^33^. Thus, there is an urgent need for developing new adjuvant anti-thrombotic therapies for AIS patients. The present study demonstrated the following: (1) PI3Kβ protein levels in platelets were significantly elevated at 24 h after tMCAO and (2) inhibition of PI3Kβ with its selective inhibitor TGX-221 administered immediately before the recanalization of the occluded MCA significantly reduced infarct volume and improved neurological function without increased risk of bleeding at 3 days after stroke. Further, we demonstrated that PI3Kβ inhibition with TGX-221 profoundly reduced downstream microvascular thrombosis and improved microvascular reperfusion, and mechanistically, these effects appear to be mediated by inhibition of circulating platelet activation and aggregation and platelet-leukocyte (neutrophil, monocyte) aggregation by flow cytometry analysis of whole blood at 24 h after stroke. These findings support an important role for PI3Kβ in mediating platelet thrombotic activity and secondary microvascular thrombosis elicited by focal cerebral I/R. While PI3Kβ is ubiquitously expressed in different types of cells, it is predominantly found in platelets. We demonstrated that the expression of PI3Kβ was detected readily in neuron-like cells with comparable levels between the normal and ischemic rat cortex (Suppl. Fig. S1). It is worth to note that we did not observe immunopositive signals in other cell types either in the normal and ischemic brain. Thus, the present study focused on understanding the changes of PI3Kβ expression in platelets and showed that PI3Kβ plays an important role in platelet activation and downstream microvascular thrombosis following cerebral I/R injury. Nevertheless, some interesting questions remain for further investigation: 1) if and how cerebral I/R activates platelet PI3Kβ, 2) if and how PI3Kβ expressed by other types of cells in the brain and the periphery contribute to cerebral I/R injury, and 3) if and how therapeutic blocking PI3kβ can improve long-term neurological outcomes after cerebral I/R injury.

PI3Kβ has emerged as a new target for antithrombotic therapy^12^. Among the class I PI3Ks (p110α, -β, -γ, -δ) that all are expressed in platelets, PI3Kβ is the most abundant isoform in platelets and plays a dominant role in regulating platelet adhesion and aggregation^34^. With either PI3Kβ inhibitors^35^ and PI3Kβ deficient mice^36,37^, previous studies have shown that PI3Kβ regulates platelet aggregation and thrombosis mainly through modulating signals downstream of the Gi-coupled P2Y12 receptor and sustained integrin αIIbβ3 activation. Integrin αIIbβ3 is a major membrane protein expressed on the surface of platelets and plays a central role in normal hemostasis and pathological thrombosis. On unactivated platelets, αIIbβ3 has little affinity for soluble fibrinogen^38^. Platelet activation leads to activation of αIIbβ3 through inside-out signaling, resulting in a significant increase in the affinity for fibrinogen^38^. On stimulated platelets, αIIbβ3 serves as a specific receptor for fibrinogen (and fibrin). Fibrinogen is converted into the insoluble protein fibrin during coagulation. Platelet aggregation and thrombus formation is determined by the integrin αIIbβ3-mediated interactions of platelets with fibrinogen (and fibrin), presumably a fibrinogen molecule acts as a bridge between αIIbβ3 molecules on different platelets^38^. PI3Kβ inhibitors undermine stable platelet aggregation in vitro, and in vivo these inhibitors eliminate occlusive thrombus formation but do not prolong bleeding time, affording protection from thrombotic occlusion^12,37^. The present study demonstrated that PI3Kβ inhibition with TGX221 significantly reduced circulating platelet activation (determined by platelet P-selectin expression) and platelet aggregation (assessed by platelet-fibrinogen binding assay) after I/R, and ADP-induced platelet aggregation in vitro. These findings support an important role for PI3Kβ in mediating thrombotic activity of platelets, a key contributing factor to secondary microvascular thrombosis after I/R injury. As a consequence, PI3Kβ inhibition with TGX-221 profoundly reduced the deposition of platelets and fibrin in the cerebral microvessels after I/R.

Elevated levels of platelet activation and platelet-leukocyte aggregates contribute importantly to inflammatory and thrombotic events in ischemic stroke^39^. Experimental studies have shown that microvascular lumina were obstructed with platelets, leukocytes, and fibrin-rich aggregates during early I/R^40–42^. Thus, preventing secondary microvascular thrombosis should improve microvascular reperfusion in stroke^18^. It has been widely recognized that the formation of platelet-leukocyte aggregates is mediated via platelet P-selectin binding to P-selectin glycoprotein ligand 1 (PSGL-1) on the leukocyte^43^. The present study demonstrated that inhibition of PI3Kβ with TGX-221 profoundly inhibited circulating platelet P-selectin expression and reduced circulating platelet-leukocyte (neutrophil, monocyte) aggregates elicited by tMCAO.

Thromboxane A2 (TxA2) is a member of the family of lipids known as eicosanoids. It is produced mainly by activated platelets via a cyclooxygenase (COX) enzyme in the platelet cytosol^44^. TxA2 synthesis by platelets increases in response to a variety of stimuli (e.g. thrombin, collagen, and ADP) during the times of tissue injury. The released TxA2 acts as a positive feedback mediator that stimulates platelet activation and promotes the recruitment of more platelets into growing thrombi, thereby contributing to the pathophysiology of thrombus formation in ischemic stroke^44^. TxA2 is highly unstable, with a half-life of about 30 s, and rapidly hydrated to thromboxane B2 (TxB2), an inactive stable metabolite). PI3Kβ has been shown to mediate ADP-induced TxA2 generation by activated platelets in vitro^45^. In this study, we measured TxB2 instead of TxA2 and found that blocking of PI3Kβ with TGX221 significantly decreased plasma TxB2 levels after stroke.

In summary, our study shows that increased platelet expression of PI3Kβ is associated with platelet activation and aggregation, and downstream microvascular thrombosis after ischemic stroke, and blocking of PI3Kβ has the potential to protect against I/R injury in acute ischemic stroke.

## Supplementary Information

Supplementary Figures.

## Data Availability

All relevant data are within the paper.
