# Supplementary material for: Blocking of PI3-kinase beta protects against cerebral ischemia/reperfusion injury by reducing platelet activation and downstream microvascular thrombosis in rats
Source: Sci Rep. 2023 Feb 4;13:2030. doi: 10.1038/s41598-023-29235-2 (PMC9899241; doi:10.1038/s41598-023-29235-2)
Supplement: Supplementary file 1 — Supplementary Figures. [file 41598_2023_29235_MOESM1_ESM.pdf]

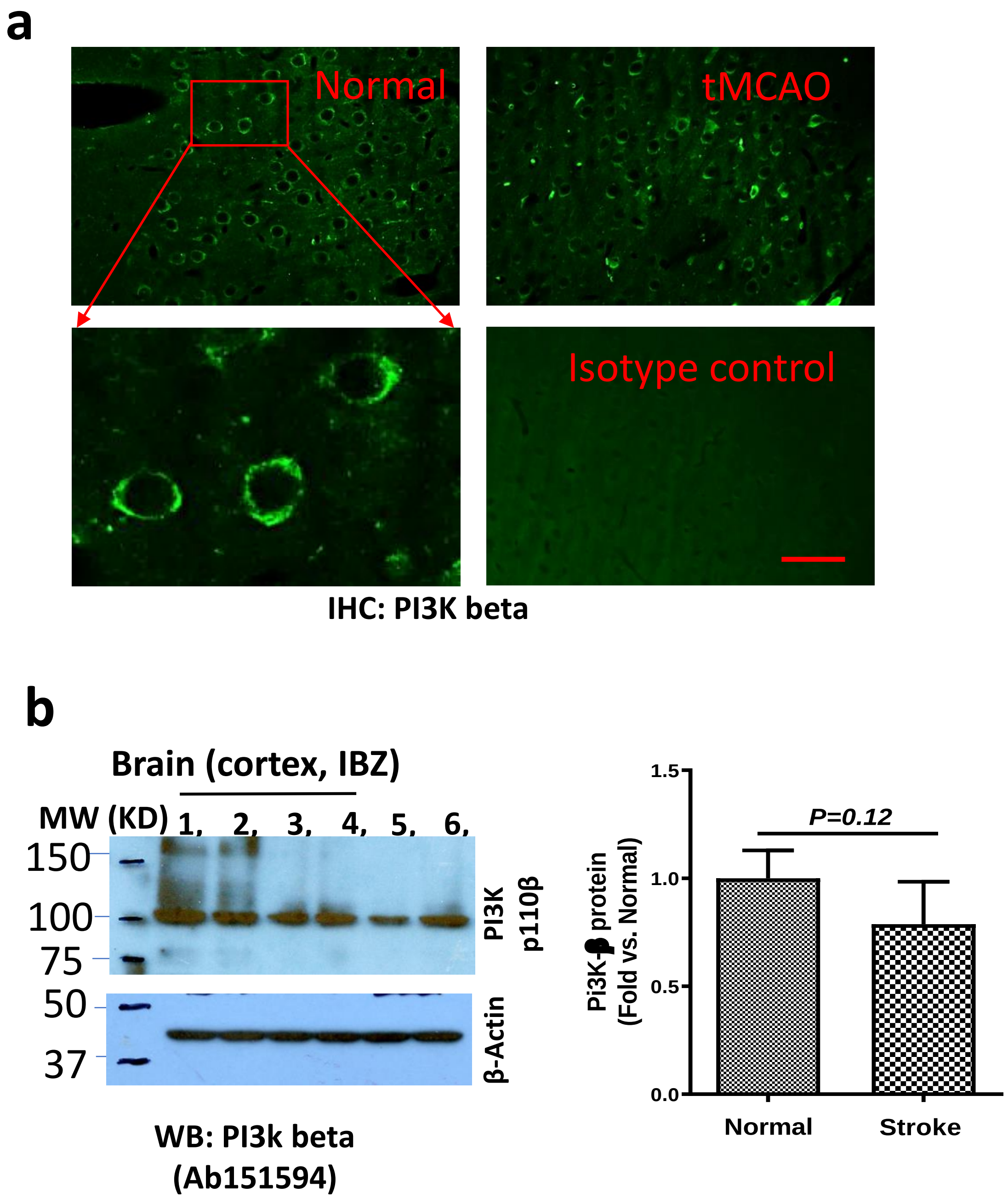

Fig. S1, PI3K-β expression in the brain. **a,** Representative IHC staining (≈ -1.0 mm to bregma) showing the expression of PI3K-β on the neuron-like cells in the brain cortex in normal and ischemic stroke animals. The regions of IBZ were photographed. Bar=50 µm. N=3/group. Animals were euthanized 24 hours after stroke. **b,** Representative images of Western blots showing PI3K-β protein levels using protein extracts from cortical ischemic boundary zone (IBZ) or corresponding regions in normal brain. Animals were euthanized 24 hours after stroke. Line 1, 2: normal brain tissue samples; Line 3, 4: ischemic brain tissue samples. Line 5, 6: B35 cells (a rat neuronal neuroblast cell line, used as control). The data showed that the basal levels of PI3K-β protein in the normal cortex were readily detectable and they were slightly decreased after stroke. N=4/group. β-actin was used for loading control. Molecular marker: all blue standards, bio rad:161-0393.

Supplement Figure S1

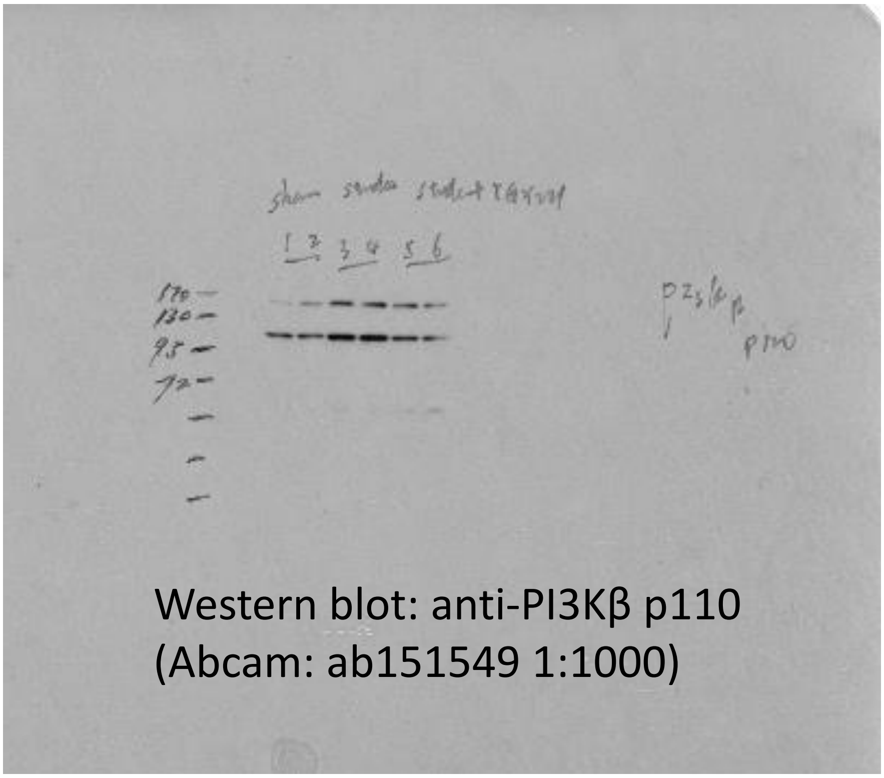

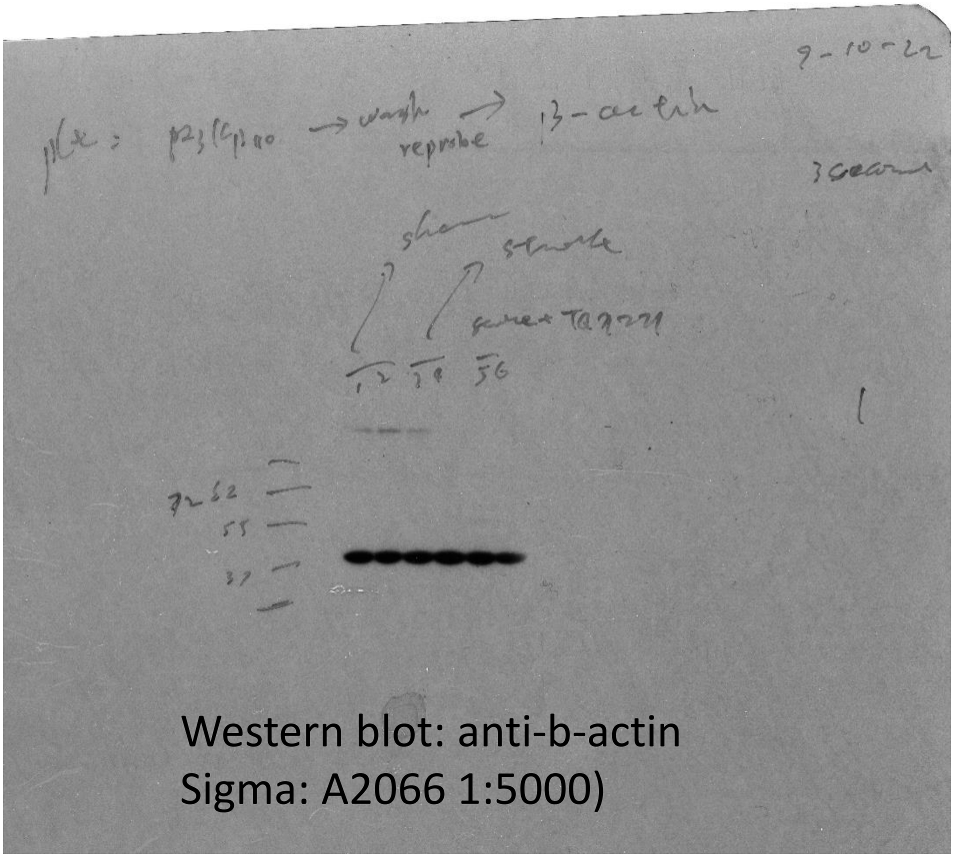

Supple Figure 2: Original images of western blot in Fig 3A.

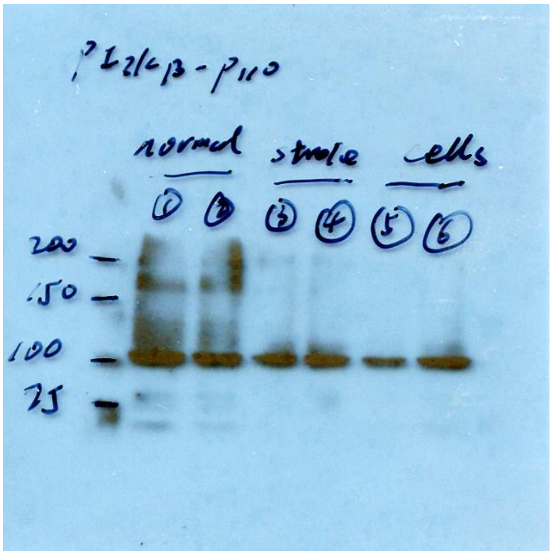

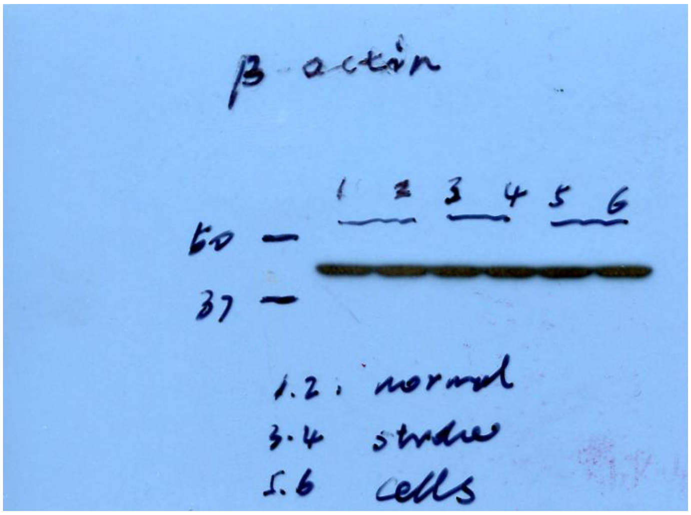

Supple Figure 3: Original images of Western blot for Pi3k beta and b-actin in Suple.1
